# Synergistic Neuroprotective Effects of a Natural Product Mixture against AD Hallmarks and Cognitive Decline in *Caenorhabditis elegans* and an SAMP8 Mice Model

**DOI:** 10.3390/nu13072411

**Published:** 2021-07-14

**Authors:** Christian Griñán-Ferré, Aina Bellver-Sanchis, Mónica Olivares-Martín, Oscar Bañuelos-Hortigüela, Mercè Pallàs

**Affiliations:** 1 Pharmacology Section, Department of Pharmacology, Toxicology and Therapeutic Chemistry, Faculty of Pharmacy and Food Sciences, Institute of Neuroscience, University of Barcelona (NeuroUB), Av. Joan XXIII 27-31, 08028 Barcelona, Spain; abellversanchis@gmail.com; 2 R&D Department, Biosearch S.A. Camino de Purchil 66, 18004 Granada, Spain; molivares@biosearchlife.com (M.O.-M.); obanuelos@biosearchlife.com (O.B.-H.)

**Keywords:** aging, AD hallmarks, Alzheimer’s disease, nutritional intervention, natural extracts, *C. elegans*, SAMP8

## Abstract

The study of different natural products can provide a wealth of bioactive compounds, and more interestingly, their combination can exert a new strategy for several neurodegenerative diseases with major public health importance, such as Alzheimer’s disease (AD). Here, we investigated the synergistic neuroprotective effects of a mixed extract composed of docosahexaenoic acid, *Ginkgo biloba*, D-pinitol, and ursolic acid in several transgenic *Caenorhabditis elegans (C. elegans)* and a senescence-accelerated prone mice 8 (SAMP8) model. First, we found a significantly higher survival percentage in the *C. elegans* group treated with the natural product mixture compared to the single extract-treated groups. Likewise, we found a significantly increased lifespan in group of *C. elegans* treated with the natural product mixture compared to the other groups, suggesting synergistic effects. Remarkably, we determined a significant reduction in Aβ plaque accumulation in the group of *C. elegans* treated with the natural product mixture compared to the other groups, confirming synergy. Finally, we demonstrated better cognitive performance in the group treated with the natural product mixture in both AD models (neuronal Aβ *C. elegans* strain CL2355 and the SAMP8 mice model), confirming the molecular results and unraveling the synergist effects of this combination. Therefore, our results proved the potential of this new natural product mixture for AD therapeutic strategies.

## 1. Introduction

Alzheimer’s disease (AD) is known as a neurodegenerative disorder with major impacts among the elderly, being the most common form of dementia [1], and is estimated to affect 131 million people by 2050 [2]. The key common pathological hallmarks of AD include the deposition of amyloid-β (Aβ) and neurofibrillary tangles (NFTs), leading to a progressive decline in cognitive function and memory loss [3]. Nevertheless, the pathogenesis of AD appears to be complex and multifactorial, in which incidence increases with age, and age is the most important risk factor for the development of the disease. This fact promotes more than one pathological factor [4], such as oxidative stress (OS) [5] and short life expectancy [6], among others.

Extensive studies conducted in vitro and in vivo support a direct link between OS and AD pathology [6,7,8], involving Aß-induced synaptic dysfunction [5], neuronal death [9], and neurotransmitter deficits [10], highlighting its critical role in cognitive decline [11,12]. OS is caused by the overproduction of reactive oxidative species (ROS), which can damage the central nervous system (CNS) through oxygen modification of macromolecules such as lipids, proteins, and nucleic acids [13]. This increase of ROS has been associated with the age-dependent reduction of antioxidant enzymes [14], resulting in altered synaptic activity and neurotransmission in neurons leading to cognitive dysfunction. Accordingly, evidence reports that the accumulation of Aβ can exacerbate mitochondrial dysfunction and ROS production, contributing to a vicious cycle [15]. 

Despite extensive pharmacotherapeutic research regarding AD treatments, even with the recent approval of aducanumab [16], none of the approved approaches are entirely successful, leading to new approaches in lifestyle modification and its potential to prevent/delay age-related cognitive decline. In particular, greater emphasis has been placed on implementing non-pharmacological interventions that might prevent AD or reduce the progression of the disease [17,18]. Indeed, the roles of lifestyle interventions and nutraceuticals in preventing many neurodegenerative diseases are highly appreciated in the literature, and are especially relevant for AD [19].

Growing evidence has identified many natural products that can delay the aging process or the onset of age-related disease, including cognitive decline [20]. In this regard, in vivo studies have investigated and adopted several natural products such as *Ginkgo biloba* (Gb) [21,22,23], D-pinitol [24], ursolic acid (UA) [25,26,27], and docosahexaenoic acid (DHA) [28,29,30], among others, for their beneficial effects against AD through the modulation of different molecular events. Thus, findings from in vitro studies follow the same line, reporting modulations in events critical for AD [31,32,33,34]. For instance, numerous studies have described that the beneficial action of Gb is mainly due to its free radical scavenging action, as it effectively attenuates oxidative damage triggered by H_2_O_2_/FeSO_4_ in brain granule cells [35,36]. Moreover, extensive clinical trials with the aforementioned dietary compounds are ongoing [37,38,39,40], although studies and more extended intervention periods are necessary to define optimal dosages. Thus, despite natural extracts being widely used and evaluated, their efficacy for AD’s preventive or curative strategy remains controversial, in that most of these trials do not support robust clinical effects for AD. Interestingly, combinations of different active ingredients in extracts might lead to additive or synergistic effects, giving better disease-modifying activity [41,42]. Studies evaluating the synergistic effects of the above natural compounds for the treatment of AD are lacking. Hence, the aim of this work follows the hypothesis that the combination of several extracts [43,44] may promote synergistic effects and pave the way to quick and better activity regarding cognitive decline and AD hallmarks with minimal side effects.

For this purpose, we used *Caenorhabditis elegans (C. elegans)* as an in vitro model organism and the senescence-accelerated mouse prone 8 (SAMP8) model to evaluate the neuroprotective role of a natural product mixture composed of Gb 50.4 mg/L, DHA 49.3 mg/L, D-pinitol 20.4 mg/L, and UA 16 mg/L. *C. elegans* has emerged as a powerful tool to study the underlying mechanisms that give rise to aging-associated neurodegenerative diseases [45]. Thus, it allows us to determine the effects of the mixture in behavioral and molecular improvements as well as modulations in lifespan. We used the transgenic strain CL2006, which expresses human Aβ_1-42_ under control of a muscle-specific promoter and responds to Aβ_1-42_ aggregation with progressive adult-onset paralysis, to evaluate the effects of the selected compounds on Aβ toxicity [46]. Furthermore, to identify a neuronal behavioral phenotype, strain CL2355, in which Aβ is expressed in neurons, and its control, CL2122, were used [47]. 

Moreover, to evaluate whether the same effect could be observed in mammals, we used the SAMP8 mouse, a mouse model of age-related cognitive decline and late-onset AD (LOAD) [48,49]. SAMP8 is a mice model established through phenotypic selection from a common genetic pool of the AKR/J-strain of mice [50]. Besides, this strain displays behavioral abnormalities and cognitive decline [51]. Thus, features such as oxidative damage associated with mitochondrial dysfunction [52] that cause the overproduction of amyloid precursor protein (APP) processing [53] and pathological accumulations of hyperphosphorylated tau [54], which are implicated in age-dependent cognitive decline [55], made SAMP8 a suitable mouse model to evaluate the effects of the natural product mixture treatment for AD.

## 2. Materials and Methods

### 2.1. Study Design

**Figure 1:**
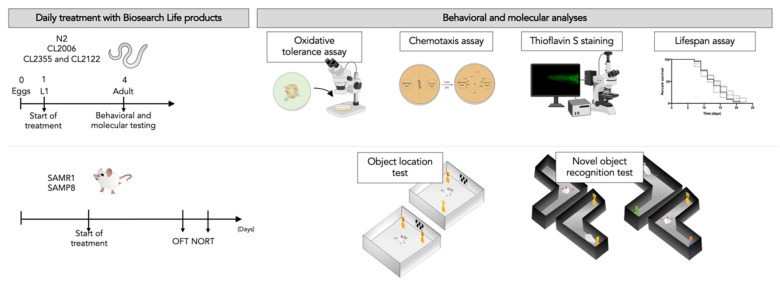
Representative experimental design with schematic behavioral and molecular studies. L: larval stage; SAMR1: Senescence-Accelerated Mouse Resistant 1; SAMP8: Senescence-accelerated mouse prone 8; OFT: Open field test; NORT: Novel object recognition test.

The overall objective of the study was to evaluate the synergistic effect of a natural product mixture. The first part of this study was to determine the beneficial effect of the product mixture on aging hallmarks in *C. elegans* through cognitive and molecular techniques. The second aim of the study was to validate resultant beneficial effects in a vertebrate model, SAMP8. For all animal experiments, we ensured blinded outcome assessment. The sample size for the intervention was chosen following previous studies in our laboratory and using one of the available interactive tools (http://www.biomath.info/power/index.html). According to each assay, we used *n* = 120–150 or 50–70 as total for at least three replicates in *C. elegans*. For mice, we used *n* = 36 for cognitive tests SAMR1 (*n* = 12), SAMP8 (*n* = 24), see Figure 1.

### 2.2. Worm Strains, Maintenance, and General Methods

Strains used in this work and abbreviations are listed in Table 1. All the strains were backcrossed to Bristol strain N2 (WT) five to twelve times. Standard methods were used for culturing and observing *C. elegans*, unless otherwise noted. Wild-type nematodes were propagated at 20 °C, while transgenic strains were maintained at 16 °C in a temperature-controlled incubator on solid nematode growth medium (NGM) seeded with *Escherichia coli (E. coli)* OP50 strain as a food source. To obtain the age synchronized population of eggs, gravid adults were treated with an alkaline hypochlorite solution (0.5 M NaOH, ∼2.6% NaClO) for 5–7 min. Fertilized eggs were suspended in S-medium for 12 h and L1 larvae were allowed to hatch overnight in the absence of food. 

### 2.3. Mice and Maintenance 

Seven-month-old SAMP8 (*n* = 24) and SAMR1 (*n* = 12) mice were used to perform the behavioral experiments. The animals were divided randomly into three groups: SAMR1 control (*n* = 12), SAMP8 control (*n* = 12), and SAMP8 treated with the natural product mixture extract (SAMP8 Mix) (*n* = 12). The animals had free access to food and water under standard temperature conditions (22 ± 2 °C) and 12h:12h light-dark cycles (300 lux/0 lux). All studies were performed in accordance with the Institutional Guidelines for the Care and Use of Laboratory Animals established by the Ethical Committee for Animal Experimentation at the University of Barcelona.

### 2.4. Compound Preparation and Treatments

All vegetal extracts and natural products were provided by Biosearch life (Granada, Spain), and were used without further purification. The proportion of each extract in the natural product mixture was as follows: 38% Gb, 36% DHA, 15% D-pinitol, and 11% UA.

For *C. elegans*, extracts were serially diluted in a range between 10 g/L and 1.6 g/L in 100% dimethyl sulfoxide (DMSO, Sigma Aldrich, St. Louis, MO, USA). Each concentration was then diluted in MilliQ purified water to obtain a final concentration ranging between 100 mg/L and 16 mg/L in 1% DMSO in well. The natural product mixture was composed of 50.4 mg/L Gb, 49.3 mg/L DHA, 20.4 mg/L D-pinitol, and 16 mg/L ursolic acid-rich extract (Ursolia^®, Biosearch life^, Granada, Spain), maintaining the previously established proportions. In most cases, treatments were performed in liquid culture for 4 days at 20 °C. Each well contained a final volume of 60 µL, comprising 25–30 animals in the L1 stage, products under study at the appropriate doses, and OP50 inactivated by freeze-thaw cycles and suspended in S-medium complete to a final optical density = 595 (OD_595_) of 0.9–0.8 measured in the microplate reader (Benchmark™ Plus Microplate Reader, BioRad, Hercules, CA, USA). For the chemotaxis assay, synchronized CL2355 and its control CL2122 were treated with products on fresh NGM plates seeded with inactivated *E. coli,* starting from the L1 stage. They were cultured at 16 °C for 36 h, and then at 23 °C for another 36 h.

For mice, the natural product mixture was dissolved in 1.8% 2-hydroxypropyl-β-cyclodextrin and administered through drinking water for 4 weeks. Water consumption was controlled each week.

### 2.5. C. elegans: Oxidative Tolerance Assay

Treated, adult, wild-type worms (N2) were transferred onto plates that included 6.2 mM t-butyl hydroperoxide (CAS# 75-91-2, Alfa Aesar, Kandel, Germany) in NGM agar. The worms were incubated on these plates at 20 °C for 2 h. Then, the worms were transferred to new NGM plates seeded with OP50, and without t-butyl hydroperoxide. The worms were observed 2 h, 24 h, and 48 h after intervention and scored as dead when they did not respond to repeated prodding with a pick. As a positive control, 58 µM vitamin C (L-(+)-Ascorbic acid 99%, CAS# 50-81-7, Alfa Aesar, Kandel, Germany) were used.

### 2.6. C. elegans: Chemotaxis Assay

Nematodes were collected after their respective treatments and washed with M9. Briefly, the assay was performed in 100 mm NGM plates, 10 µL of odorant (0.5% benzaldehyde in 96% ethanol) (CAS# 100-52-7, Sigma Aldrich, St. Louis, MO, USA), along with 1 M of sodium azide (CAS# 26628-22-8, Sigma Aldrich, St. Louis, MO, USA) added to the “attractant” spot. On the opposite side, 10 µL of control odorant (96% ethanol) along with 1 M of sodium azide were added. Immediately after, 50–60 worms were placed towards the center of the plate. Assay plates were incubated at 23 °C for 1 h and were scored according to the chemotaxis index (CI) as follows: CI = (number of worms at attractant−number of worms at control)/total number of worms. In each experiment, at least 60 worms from each group were analyzed. 

### 2.7. C. elegans: Thioflavin-S Staining Aß Aggregation

Age-synchronized CL2006 worms were fixed in 4% paraformaldehyde/PBS, pH 7.5, for 24 h at 4 °C, and permeabilized in 5% fresh β-mercaptoethanol, 1% Triton X-100, 125 mm Tris, pH 7.5, at 37 °C for another 24 h. The nematodes were stained with 0.125% Thioflavin S (ThS) (CAS# 1326-12-1, Sigma Aldrich, St Louis, MO, USA) in 50% ethanol for 2 min, de-stained in 50% EtOH for 2 min, washed three times with PBS, and transferred in approximately 10 µL volume on a droplet of Fluoromount G (CAT#17984-25, Electron Microscopy Sciences, Hatfield, PA, USA) on a glass slide for microscopy. Fluorescence images were acquired using a 20Å~ objective of a fluorescence microscope. Amyloid deposits in the head regions of the worms were quantified by counting the number of ThS-positive spots using ImageJ, and were expressed as Aß deposits/anterior area. 

### 2.8. C. elegans: Lifespan Assay

Worms were treated as described above in liquid culture for four days, starting at the L1 stage. However, to prevent progeny production, 1µL of 5′-fluorodeoxyuridine (FUdR, CAS# 50-91-9, Apollo scientific, Stockport, UK), at a final concentration of 120 µM, was added at day 4. After treatment, approximately 30 young adult worms were placed on three different NGM plates per condition and transferred to freshly seeded plates every three days, scoring dead animals. An animal was considered dead if no mechanical response was elicited upon three light touches on the head with a platinum wire.

### 2.9. SAMP8 Mice: Novel Object Recognition Test

The novel object recognition test (NORT) protocol employed was a modification of [51,56]. Briefly, mice were placed in a 90°, two-arm, 25-cm-long, 20-cm-high, 5-cm-wide black maze. Before performing the test, the mice were individually habituated to the apparatus for 10 min over three days. On day four, the animals were allowed to explore freely a 10 min acquisition trial (first trial), during which they were placed in the maze in the presence of two identical, novel objects at the end of each arm. After a delay (2 h and 24 h), the animal was allowed to explore two objects one old object and one novel object. The time that mice explored the novel object (TN) and time that mice explored the old object (TO) were measured. A discrimination index (DI) was defined as (TN−TO)/(TN+TO). Exploration of an object was defined as pointing the nose towards at a distance ≤2 cm and/or touching it with the nose. Turning or sitting around the object was not considered exploration. To avoid object preference bias, objects were counterbalanced.

### 2.10. SAMP8 Mice: Object Location Test

The object location test (OLT) is a well-established task based on the spontaneous tendency of rodents to spend more time exploring a novel object location than a familiar object location, and recognizing when an object has been relocated [57,58]. Briefly, the test was performed over three days in a wooden box (50 cm *×* 50 cm *×* 25 cm), in which three walls were white except one that was black. The first day, the box was empty, and the animals just habituated to the open field arena for 10 m in. The second day, two objects were placed in front of the black wall, equidistant from each other and the wall. The objects were 10 cm high and identical. The animals were placed into the open field arena and allowed to explore both objects and surroundings for 10 min. Afterward, animals were returned to their home cages, and the OLT apparatus was cleaned with 70% EtOH. The third day, one object was moved in front of the white wall to test spatial memory. Trials were recorded using a camera mounted above the open field area, and the total exploration time was determined by scoring the amount of time (seconds) spent sniffing the object in the new location (TN) and the object in the old location (TO). To evaluate the cognitive performance, the DI was calculated, which is defined as (TN-TO)/(TN+TO).

### 2.11. Statistics

All statistical analysis was conducted using GraphPad Prism GraphPad Prism ver. 9 statistical software. Groups were compared with one-way analysis of variance (ANOVA), followed by the Tukey post hoc test. In some cases, comparison between groups was also performed by two-tailed student’s t-test for independent replicates. For the further statistical analysis of *C. elegans* survival assay, the Kaplan–Meier survival plot was constructed, which illustrates the percentage of live animals against time. Data are expressed as the mean ± standard error of the mean (SEM) of at least *n* = 3. Statistical significance was considered when *p* values were <0.05. The statistical outliers were determined with Grubs’ test and removed from the analysis.

## 3. Results

### 3.1. Natural Product Mixture Attenuates Oxidative Stress in C. elegans

**Figure 2:**
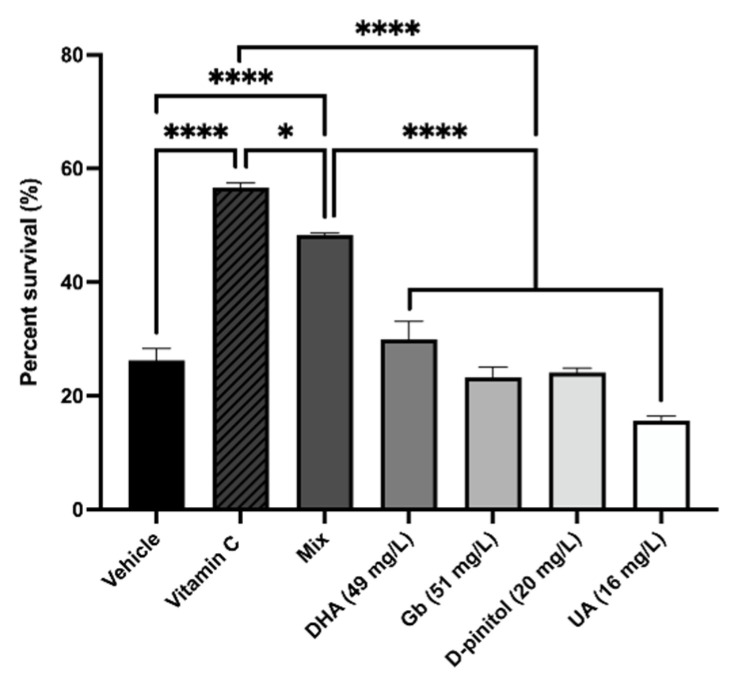
Summary of the OS response in different *C. elegans* N2 (WT) groups after treatment with each extract treatment, natural product mixture, or vitamin C (58 µM). Values represented are mean ± standard error of the mean (SEM); *n* = 3 with 120–150 worms in each group. * *p* < 0.05; **** *p* < 0.0001. DHA: docosahexaenoic acid; Gb: *Ginkgo biloba*; UA: ursolic acid.

To investigate if the natural products and mixture have any beneficial effects on OS, we tested them after exposure to the chemical oxidant Tert-butyl (6.2 mM). We found that each product group did not reach protection against OS in comparison with the untreated control. Additionally, we obtained a significant reduction in the percent survival in comparison with the vitamin C group (Figure 2). On the other hand, the worms pre-treated with the natural product mixture (dose) were significantly protected against tert-butyl (6.2 mM)-induced OS in comparison with the untreated group, and reached the percent of survival worms close to the vitamin C group (58 µM), demonstrating the synergistic effect of the combined administration of the products (Figure 2). 

### 3.2. Mean Lifespan Extension by Natural Product Mixture in C. elegans

**Figure 3:**
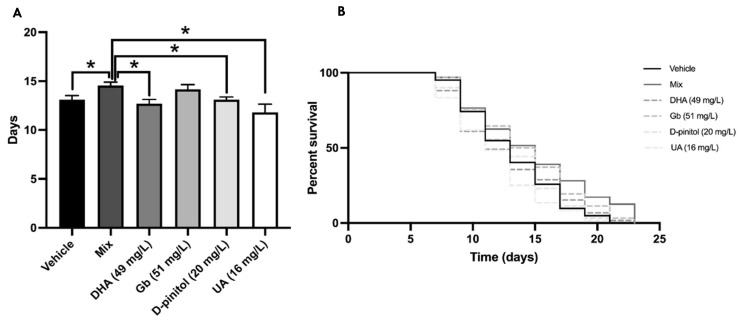
Kaplan–Meier curve for the survival of C. elegans on different extracts (**A**). The lifespan means of the C. elegans treated and control groups with DMSO 1% (**B**). Values represented are mean ± standard error of the mean (SEM); *n* = 3 with 60–70 worms in each group. * *p* < 0.05. DHA: docosahexaenoic acid; Gb: *Ginkgo biloba*; UA: ursolic acid.

Further, we studied the effects by examining changes in lifespan in *C. elegans* after different natural product treatments and mixtures. Firstly, we did not find any significant change among groups with use of the Kaplan–Meier curve (Figure 3B). However, the mean lifespan was extended by up to 15% with the natural product mixture treatment in comparison with the DHA, D-pinitol, and UA groups, demonstrating synergistic effect (Figure 3A), whereas there were no changes between the mixture and Gb group due to the well-described effects of Gb on lifespan. 

### 3.3. Natural Product Mixture Suppresses Neuronal Aβ Expression-Induced Defectd in Chemotaxis Behavior in Transgenic C. elegans (CL2355)

**Figure 4:**
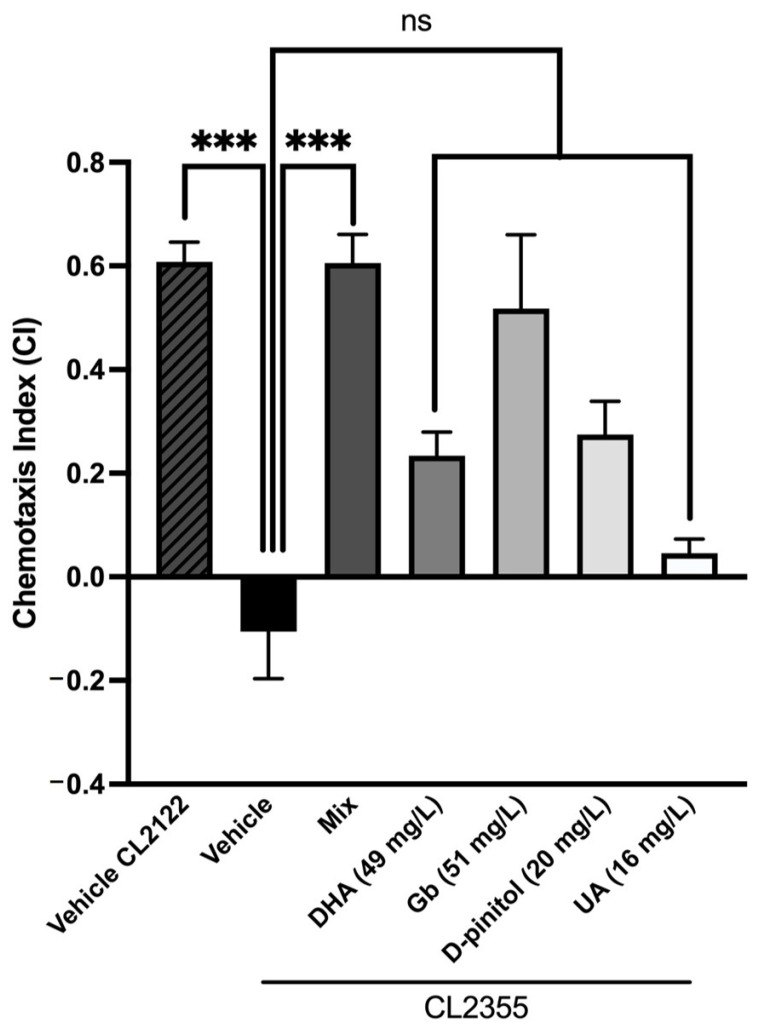
Chemotaxis assay results in the neuronal Aβ strain CL2355. Values represented are mean ± standard error of the mean (SEM); *n* = 3 with 120–150 worms in each group. *** *p* < 0.001; ns: non-significant. DHA: docosahexaenoic acid; Gb: *Ginkgo biloba*; UA: ursolic acid.

We used a transgenic strain, CL2355, to investigate whether the inhibitory effects of the natural product mixture on Aβ oligomerization would protect neurons against Aβ. The chemotaxis index (CI) was scored for all groups on day 5 and is a measure of the fraction of worms that are able to arrive at the attractant side. Figure 4 shows that the CL2355 strain exhibits a significant reduction in the CI compared to the control strain CL2122. Moreover, each natural product when separated shows a slight tendency to increase the CI compared to the CL2355 strain, but not significantly. Interestingly, a significant increase was observed in the CI in the mixture-treated group compared to the CL2355 group, demonstrating the synergistic effects and restoring chemotaxis behavior to the control strain CL2122 (Figure 4). These results suggest that the natural product mixture might protect neurons against Aβ-induced toxicity and rescue neuronal Aβ expression-induced defects in chemotaxis behavior.

### 3.4. Natural Product Mixture Improves Amyloid-ß Burden in Transgenic C. elegans (CL2006)

**Figure 5:**
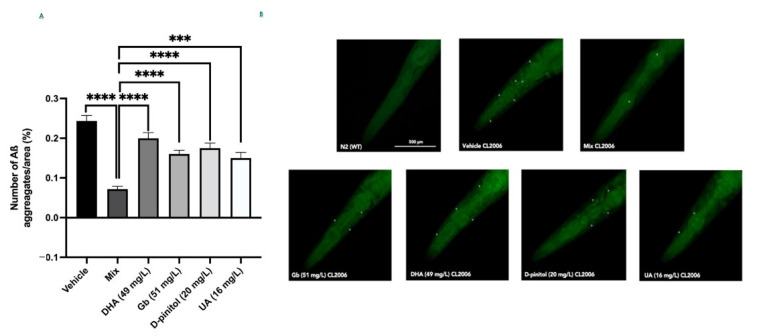
Quantification of ThS-positive particles in the head region of the CL2006 strain (**A**). Representative images from each group tested (**B**). Values represented are mean ± standard error of the mean (SEM); *n* = 4 with 50–60 worms in each group. **** *p* < 0.0001. DHA: docosahexaenoic acid; Gb: *Ginkgo biloba*; UA: ursolic acid.

Next, we scored the number of Aß deposits in the head of the *C. elegans* strain CL2006 to evaluate whether the inhibitory effects of the mixture versus each individual compound on Aβ oligomerization would reduce the degree of amyloidosis. In addition, Figure 5B respectively shows Aβ deposits (white arrows) detected in CL2006 but not in the wild-type (N2). Figure 5A shows that the mixture group had a remarkable effect on the deposits of the Aß1-42 peptide, suggesting that this composition could decrease the aggregation of Aß species significantly in a synergistic way. However, the different product groups (DHA, Gb, D-pinitol, and UA) did not have any effect on the deposits of the Aß1-42, demonstrating that the synergistic effect obtained in the mixture group is not apparent (Figure 5A). 

### 3.5. Product Mixture Improves Cognitive Decline Presented by the SAMP8 Mice Model

**Figure 6:**
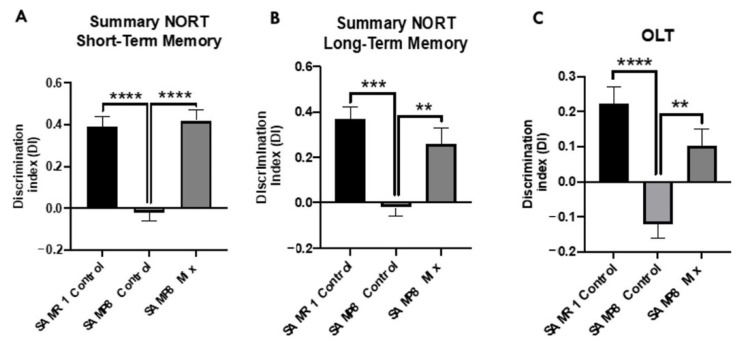
Results of the discrimination index (DI) of short-term memory (**A**), and long-term memory (**B**), from novel object recognition tests (NORT) in all Senescence-accelerated mouse prone 8; (SAMP8) groups. Results of the DI from object location test (OLT) (**C**). Values represented are mean ± standard error of the mean (SEM); *n* = 36 (SAMR1 control *n* = 12; SAMP8 control *n* = 12; SAMP8 natural product mixture *n* = 12). * *p* < 0.05; ** *p* < 0.01; *** *p* < 0.001; **** *p* < 0.0001. SAMR1: Senescence-Accelerated Mouse Resistant 1.

We evaluated the beneficial effects on SAMP8 cognition after treatment with the natural product mixture through NORT and OLT. NORT evaluation demonstrates the cognitive improvement of the SAMP8 treatment group through both short- and long-term memory tests in comparison with the SAMP8 control group, reaching DI levels of the SAMR1 control group (Figure 6A,B). Regarding the OLT evaluation, significantly higher DI values were observed in the SAMP8 natural product mixture group compared to the SAMP8 control, recovering DI levels to those of the SAMR1 control group (Figure 6C). Thus, both results showed better cognitive performance in the SAMP8 treated with natural products, suggesting the neuroprotective effects of the natural product mixture. 

## 4. Discussion

Although the controversial aducanumab was recently Food and Drug Administration (FDA) approved for the treatment of AD [16], there is still no cure for age-related cognitive decline and AD [59,60]. Natural compounds that exert several beneficial properties through different molecular pathways could represent a promising strategy to prevent or cure age-related cognitive decline and AD risk [61]. Indeed, a wealth of natural extracts that can relieve AD symptoms have been identified according to different targets and mechanisms. Although some of them have entered clinical trials, most are still in the subclinical trial phase, and further evidence is needed to prove the efficacy of these extracts in clinically relevant animal models of AD [62]. Interestingly, a growing interest in the synergism between/among different natural compounds targeting Aβ toxicity in AD opens up a new insight into non-pharmacological AD research [63,64].

Our main goal was to demonstrate that combining different natural products with similar biological effects increases therapeutic efficacy in AD in comparison to single extract treatments. Such potentiation effects were produced by using a submaximal or minimal concentration of 38% DHA, 36% Gb, 15% Pinitol, and 11% UA, which, according to the literature, have not shown any protective effect by themselves, obtaining the proposed composition of the mixture. In this study, we evaluated the synergistic neuroprotective effects of the composition against AD hallmarks and cognitive decline using two approaches: *C. elegans* and an AD murine model. The intervention ended on the fifth day for *C. elegans* when they reached the adult stage, and thereby is a chronic treatment. On the other hand, the SAMP8 intervention was finished when the animals reached seven months old, when cognitive impairment and remarkable pathological similarities to AD are presented, such as OS and aberrant amyloid processing pathway, among others [65].

Several causal mechanisms of brain aging-related impairment have been proposed, and one of the relevant events is OS, which is associated with the etiology of age-related cognitive decline and AD [66]. Thus, it has been reported that OS promotes senescence [67,68], and a shortened lifespan in worms [69,70]. Accordingly, similar results were also observed in mice [68]. Considering this evidence, we assessed oxidative tolerance assay in the *C. elegans* WT strain and observed the treatment effect on lifespan up to 48 h after exposure to Tert-butyl. Of interest, we found that the natural product mixture treatment improved the survival percentage significantly compared to the rest of groups treated with single natural compounds, demonstrating the synergistic protective effects of the composition. 

As previously mentioned, OS occurs early in the progression of the neurodegenerative process presented by AD, supporting its role in the disease [71], and interestingly it is linked to the presence of Aβ plaques [72]. Furthermore, the association between the amounts of Aβ plaques and cognitive decline is highly correlated [73,74]. Here, we demonstrated that the natural product mixture exerted a reduction in Aβ plaques presented by the transgenic *C. elegans* strain CL2006, whereas the rest of the groups treated with single compounds showed a slight tendency to reduce Aβ plaques. These results demonstrated again that there was no evidence to suggest this result at those doses. For instance, our natural product mixture contained a dose of 51 µg/mL of Gb, whereas Wu et al. [23] showed that the inhibition of Aβ oligomerization and Aβ deposits in worms was reached at the dose of 100 µg/mL of Gb (EGb 761), strengthening the synergism-enhancing effects of the mixture produced when using a submaximal dose.

Considering that aging is the main risk factor of AD [23], we evaluated lifespan in *C. elegans* after treatment with the product mixture. Our study found an extended lifespan by up to 15% compared to the other groups, except in the Gb group, whose beneficial effects on lifespan are well established at 100 µg/mL [75]. Following our results, no lifespan effects were described, for neither D-pinitol nor DHA at these doses [76]. Additionally, it has been reported that UA prolongs the lifespan of *C. elegans* at concentrations of 100 µM [77]. Since our product mixture has a final concentration of the active ingredient UA of approximately 16 mg/L or 5 uM, this might be insufficient. Thus, taking together these findings, we again demonstrated the synergistic effects of the mixed natural extracts [77]. Furthermore, these results linked to the OS results were better in the mix of natural extracts for exploring potential anti-aging drugs. OS has been reported to be a significant cause of aging, and antioxidants have been reported to curtail aging [78,79].

Finally, we showed that the sequence of events manifested in the transgenic worms’ behavior shares similar mechanisms with the cognitive impairment in rodents. Our study revealed that the SAMP8 mice treated with the product mixture significantly improved their cognition using chemotaxis assay and NORT. On the one hand, the absence of endogenous Aβ production in the worms offers an opportunity to directly asses the role of Aβ involvement in pathological behaviors [80]. Thus, in using the transgenic strain CL2355, which exhibits defects in chemotaxis behavior due to Aβ-induced toxicity, we found that the natural product mixture protects against the pathological phenotype presented by this transgenic strain. Notably, the chemotaxis response in *C. elegans* is mediated by the activation of several sensory neurons and interneurons to stimulate the motor neurons, being biologically relevant to Aβ-induced toxicity [81]. On the other hand, we showed that the natural product mixture chronic treatment in the SAMP8 mice model enhanced their cognitive performance. Thus, these findings supported the idea that treatment with the composition attenuated cognitive decline in both models by modulating several pathways associated with AD hallmarks. 

To sum up, our study provides new evidence that the natural product mixture can delay neurodegenerative processes through pleiotropic action, reducing Aβ and OS, promoting cognitive improvement, and increasing lifespan. Our study offers new insights for compound interventions with natural products as a therapeutic strategy to prevent or cure age-related cognitive decline and AD.

## 5. Patents

Composition for use in the treatment of cognitive disorders, U.S. Patent and Trademark Office, as national phase entry of International Patent Application No. PCT/EP2021/050270.

## Figures and Tables

**Table 1:** List of *C. elegans* strains used in this work.

Strain	Genotype	Source
N2 (Bristol)	*C. elegans* wild-type	
CL2006	dvIs2 (pCL12 (unc-54/human Abeta peptide 1–42 minigene) + rol-6(su1006))	CGC
CL2355	dvIs50 (pCL45 (snb-1::Abeta 1–42::3’ UTR(long) + mtl-2::GFP) I	CGC
CL2122	dvIs15 ((pPD30.38) unc-54(vector) + (pCL26) mtl-2::GFP)	CGC

## Data Availability

Not applicable.
